# Municipal performance management during the covid pandemic

**DOI:** 10.1177/09520767221141166

**Published:** 2022-11-22

**Authors:** Obed Pasha, Willow Jacobson

**Affiliations:** School of Government, 2331 The University of North Carolina, Chapel Hill, NC, USA

**Keywords:** Performance management, crisis management, pandemic

## Abstract

The COVID-19 pandemic brought a major shift in governmental operations, including decision-making processes. In the midst of an unprecedented crisis, public managers had to make rapid decisions within an uncertain landscape and ambiguous timeline. This exploratory study aims to understand how the pandemic impacted public managers’ behavior as they attempted to better cope with the crisis. In particular, we focus on the impact of the pandemic on performance management practices in municipal governments, examining a range of components, including data analysis and use. Our cross-sectional analysis of 103 line managers from North Carolina municipal departments finds that local government managers increased their use of performance management practices during the pandemic compared to the previous (non-pandemic) year. The increased use was amplified when existing performance information practices were established before the pandemic. These exploratory findings contribute to a better understanding of the role of performance information in decision-making during a crisis and to performance management scholarship more broadly.

In March 2020, the United States started to feel the effects of the pandemic that would challenge it for months to come. On March 11, the World Health Organization declared COVID-19 a pandemic, and on March 13, President Trump declared COVID-19 a National Emergency.^1^ Suddenly, organizations big and small had to modify their operations to respond to shifting demands and safety requirements—many halted business altogether. Since federal, state, and local governments did not have that option, the way they worked had to quickly transform. Immediate decisions had to be made within an ambiguous context and timeline. Employees who had long enjoyed consistent and stable workplace environments found themselves navigating a world of uncertainty and change.

Local governments are often the first line of response in a crisis. While COVID-19 policies were being developed at a federal and state level, local jurisdictions were faced with crucial decisions of their own. They did not have the option of stopping their work providing fundamental services to their citizens, from refuse collection to public utilities and social services.

Recent and ongoing scholarship emphasizes the importance of performance management in helping public managers make evidence-based decisions in responding to the COVID-19 crises (George, Verschuere, Wayenberg, and Zaki 2020; Hall 2021; Leoni et al., 2021). Such recommendations are in line with broader scholarship exploring the transformative implications of the pandemic at all levels of government and offering strategies to deal with its disruptions (e.g., O’Flynn 2020; Sancino, Garavaglia, Sicilia, and Braga 2020; Ahn and Wickramasinghe 2021; Ahrens and Ferry 2021). The managerial and administrative choices that were made during the turbulence and uncertainty of COVID-19 serve to provide potential empirical and theoretical insights to help public managers respond to prolonged crisis situations (Dunlop, Ongaro, and Baker 2020; Pollitt 2016).

This study contributes to the growing body of public management scholarship by examining performance management practices in municipal government during the COVID-19 pandemic. Our exploratory cross-sectional analysis of 103 line managers from a variety of functional areas in local governments examines two research questions. The first is what changes did municipalities make to the level of performance management use during the COVID-19 pandemic? The second is what factors impacted performance management changes during the pandemic? We answer these questions by using data drawn from municipalities across North Carolina—by limiting our focus to a single state, we could control for variations in state laws, cultures, policies, and economic conditions.

This article begins by reviewing the existing scholarship on the COVID-19 crisis with a focus on performance management. We first review the literature with a focus on both the context impacting this study and then more specifically on research on performance management in crisis or turbulent times. Next, we explain our methodology, followed by a presentation and subsequent discussion of our results. Our findings indicate that local governments increased their use of performance management during the pandemic. The increase was enhanced by their existing performance management practices and use. Finally, we discuss the implications of our findings, emphasizing the critical role of performance information to decision makers during a crisis and the importance of ensuring that organizations have the capacity to leverage it.

## Literature review

### Context

The COVID-19 pandemic has affected all aspects of human life. For many public organizations, the pandemic has transformed the assumptions and structures they previously operated under—from bureaucratic norms and contingency planning to emergency preparedness and political oversight (Ansell, Sørensen, and Torfing 2020; Carungu, Di Pietra, and Molinari 2021). The pandemic has also exposed institutional weaknesses and capacity gaps in dealing with such a crisis, suggesting the future challenges organizations are likely to face if they don’t rethink their traditional hierarchy (Dunlop, Ongaro, and Baker 2020).

A county child protective services official, for example, struggled to consider new ways to collect data on child abuse now that children were spending more time at home and having limited interactions with teachers, nurses, and other essential reporters of abuse. Similarly, some municipal waste management divisions had to consider altering their contracts with collection companies due to a sudden increase in residential waste as people were at home more (Hantoko et al., 2021). Parks were forced to issue physical-distancing protocols, and local libraries had to find virtual methods to continue offering services (Miller-Rushing et al., 2021). Social service agencies were besieged with welfare requests and struggled to provide food and shelter to everyone who needed it (Cohen, Russo, Campos, and Allegri 2020). Public employees frequently had to operate outside their prescribed roles and responsibilities to meet such unexpected challenges (Schuster et al., 2020).

The urgency of these issues has led to emerging research on the management implications of the COVID-19 pandemic in the public sector (e.g., Hantoko et al., 2021; Miller-Rushing et al., 2021; Sancino, Garavaglia, Sicilia, and Braga 2020). This literature suggests that the pandemic exacerbated insidious problems that already existed in public administration—such as racial inequity, resource scarcity, and a lack of public trust—and these complexities need to be confronted by public managers in the long-term (O’Flynn 2020; Sowa and Lu 2017; Sancino, Garavaglia, Sicilia, and Braga 2020).

As new issues emerged and entrenched challenges worsened, the question of how to make critical decisions was consistent. Public management scholarship has long promoted the benefit of performance management, a system of decision-making for public organizations that depends on data analysis and use, and it’s a promising tool for these unprecedented times (Hall 2021). As Dunlop, Ongaro, and Baker noted in their 2020 appeal to scholars, it is essential to study the analytical and learning capacities of public organizations during the pandemic so that we can make them stronger, more equitable, and better equipped to handle future threats.

For this research we compared use of performance management in core local government service departments within the state of North Carolina. This comparison allows for variation between jurisdictional use of performance management as well as functional departments within this one state case study.

### Performance management during turbulence

Performance management “generates performance information through strategic planning and performance measurement routines and that connects this information to decision venues, where, ideally, the information influences a range of possible decisions” (Moynihan, 2008: 5). In other words, performance management is a system that allows public managers to analyze quantitative and qualitative performance information to make evidence-based decisions that will improve overall performance (Poister, Aristigueta, and Hall 2014). Behn (2003), an often-referenced scholar, outlines the multiple reasons to measure performance. His typology, consisting of eight relatively accepted managerial uses of performance information = that includes: evaluate, control, budget, motivate, promote, celebrate, learn, and improve.

The literature identifies two competing theoretical underpinnings about the effects of performance management on organizational performance. Traditionally, these systems were implemented as mechanisms to hold public employees accountable (Poister, Pasha, and Edwards 2013). Following the expectations of principal agent theory, public employees were seen as self-interested agent who look for ways to steal, shirk, and subvert the priorities of the departmental and political leadership (Dilulio Jr 1994). The accountability-based application of performance led to various negative unintended consequences including data manipulation, cream skimming, and inequitable delivery of public services (Pasha, Kroll, and Ash 2021; Radin 2006).

As a result, public organizations are increasingly moving toward a motivation-based application of performance management system (Pasha 2018). Goal-setting theory (Wright 2003) finds that clear and challenging goals motivate employees by communicating organizational values and expectations. The feedback through performance metric help employees learn about their progress and make evidence-based decisions.

Most literature on performance management focuses on the use of performance information and its impact (Kroll 2015; Gerrish 2016; Moynihan and Pandey 2010). These studies have produced contradicting evidence surrounding the impact of implementing a performance management system. Studies such as Sun and Van Ryzin (2014), Poister, Pasha, and Edrward (2013), and Pasha (2018) show a positive impact of performance management in education, public transportation, and criminal justice. Gerrish (2016) and Koning and Heinrich (2013) demonstrate mixed and null effects in child support and social services. Other such as Dias and Maynard-Moody (2007), Soss, Fording, and Schram (2011), and Pasha, Kroll, and Ash (2021) show negative impact of these systems on equity. Still Kroll (2017) find evidence for contingency factors such as stakeholder influence, and the presence of protective institutions to mitigate negative impacts.

More recently, scholars including Ammons (2020) and Kroll and Vogel (2021) have stressed the importance of multiple organizational stakeholders in implementing effective systems. Indeed, performance management systems are most effective when they are used and appreciated by multiple organizational stakeholders including administrative executives (such as the manager), strategic policy decision makers (the board), and the operating core (those within the department). For performance measurement to move to performance management it needs to be engaged, active, and accepted by a range of critical organizational stakeholders to be truly embedded in institutional decision making (Ammons 2020; Pasha 2018; Yang and Pandey 2009).

Notwithstanding the conflicting evidence, the adoption and use of performance management systems continue to increase (Pollitt 2016). Still, relatively little is known about how these systems respond during a crisis. The ongoing pandemic has renewed scholarly interest in performance management, which can play a central role in helping public managers make better decisions in uncertain situations (George, Verschuere, Wayenberg, and Zaki 2020; Hall 2021; Mitchell et al., 2021).

In their discussion of the COVID-19 pandemic, George, Verschuere, Wayenberg, and Zaki (2020) offer comparative data analysis as a means of making well-informed decisions while dealing with the crisis. Along similar lines, Hall (2021) emphasizes the critical need for data measurement in turbulent environments to gain a better understanding of the situation and to make evidence-based decisions. But as Yang (2020) points out, it is difficult to arrive at data-informed decisions when resources are depleted, competing values must be balanced, and time is constrained. He proposes that other factors, such as “trained intuition” and “reasonableness,” be employed alongside data when making decisions. Research in other disciplines such as accounting, nonprofits, and healthcare has also put a greater emphasis on performance management as a tool to mitigate the impact that COVID-19 had on services (e.g., Kober and Thambar 2021; Huber, Gerhardt and Reilley 2021).

Empirical evidence on the use of performance management systems during a crisis is scant, contradictory, and primarily concerned with the 2008–2009 Great Recession. Jimenez (2013), for example, found that these systems only increased the perceived ability of public managers to reduce deficits, not the actual deficits. In contrast, Pasha and Poister (2017, 2019) found that public transit agencies collected more data during the Great Recession.

Previous scholarship has also presented inconsistent conclusions about the long-term effect of crisis situations on organizations. One view holds that significant events, such as the COVID-19 pandemic, inevitably lead to profound transformation (Anderson, Branch, Cooper, and Dulvy 2017; Wind, Rijkeboer, Andersson and Riper 2020). Mostly driven by external forces, such events pave the way for large-scale changes by breaking down political consensus, generating extraordinary pressure on resources, or altering the social and physical landscape of an organization (Ansell, Sørensen, and Torfing 2020; Peters, Pierre, and Randma-Liiv 2011).

Crisis often causes latent conflicts to emerge, opening the field to power struggles among various stakeholders (Peters, Pierre, and Randma-Liiv 2011), who are emboldened to push for changes that would have been unthinkable during more stable times. As noted by Ansell, Sørensen, and Torfing (2020), the kinds of turbulence caused by “surprising, inconsistent, unpredictable, and uncertain events,” like the COVID-19 pandemic, “persistently disrupt our society and challenge the public sector” (p. 949).

In contrast, institutional and path dependent perspectives expect far less transition or change. According to this view, turbulence restricts broad changes for two primary reasons (Peters, Pierre, and Randma-Liiv 2011; Thelen 2000). First, a crisis often accompanies (or causes) a severe pressure on resources, which constrains the organization’s ability to invest in fundamental changes to organizational structure, personnel, and practices (Goodin 1996; Levine 1979). Second, fundamental changes to the organization are likely to cause stress, anxiety, and confusion to employees, intensifying their resistance to change and guaranteeing its failure (Bovey and Hede 2001; Bordia et al., 2006). Proponents of this view believe that decision-making methods and associated data use do not undergo significant changes in a crisis (Thelen 2000).

Indeed, public institutions are inherently conservative arrangements of nested rules, exclusive membership, and hierarchical authority, and they tend to prefer sticking to and maintaining the status quo (Hodgson 2006; North 1990). That status quo is further supported by the unyielding power dynamic among employees, clients, and elected officials that resists fundamental shifts (Pierson 2000). In support of this assertion, Kim and Chen (2020) found that local governments in New York State repeatedly employed the same cutback strategies in dealing with multiple recession events over decades. But the question that remains unanswered is if an ongoing pandemic might be enough to change the status quo altogether.

The literature thus presents competing expectations of how performance management could change because of the pandemic. Either the COVID-19 pandemic will transform how performance management is used (e.g., Ansell, Sørensen, and Torfing 2020) or the status quo will continue (Pierson 2000). Our research attempts to elaborate on questions of performance information use during the COVID-19 pandemic by focusing on the local government setting. We seek to explore whether and to what extent municipalities changed performance management use during the COVID-19 pandemic and what factors impacted such changes.

## Methodology

This research relies on an electronic survey of municipal department directors in North Carolina. We administered the survey on 11 February 2021 and followed-up with reminders two and three weeks later on February 25 and March 4. A final request for participation was made on 23 March 2021, and the survey was closed on 25 March 2021.

### Survey measures

The survey was divided into sections that evaluated analysis, use, stakeholders, demand, and formal decision-making. Multiple survey questions measured performance information analysis and were developed by drawing on previous work by Pasha and Poister (2017) and Kroll (2015). Performance information use measures were developed by drawing on Behn’s 2003 typology of performance information objectives: to motivate, evaluate, control (hold accountable), budget, motivate, promote, celebrate, learn, and improve.

Recognizing the reality public managers faced in responding to the COVID-19 crisis the focus on promoting and celebrating were excluded from the survey. Given the timing of the survey and the extreme stress local governments were facing the researchers determined that implying that these directors should be focused on promoting and celebrating in the midst of a devastating public health crisis could be seen as insensitive.

A demand measure was used to determine the extent to which service demand changed during the pandemic. Finally, the level of formalized strategy pre-pandemic (2019) and during the pandemic (2020) years was included (Pasha and Poister 2017). The survey captures the use of performance information for decision-making by department heads in 2019 (before the pandemic), measured on a 7-point Likert scale, as well as how much that practice has changed (whether it increased or decreased) during the pandemic, measured on an 11-point slider scale (See Appendix A for survey items). This approach helps us explore change with respect to the previous year’s behavior, allowing respondents to cognitively focus on the change in level with respect to the base year. Given the perception-based nature of this data, this approach allows for greater variation and prevents the potential of anchoring level use in which respondents default to reporting the same response patterns as they had indicated for the past year.

This approach allows us to ascertain the perception of performance management use before the pandemic (i.e., the initial condition) and the perception of how it changed during the pandemic (Duit 2007; Pierson 2011). By anchoring the survey in past use, we could draw conclusions regarding relative (rather than absolute) changes from 2019 to 2020. All of the data is subjective and depends on self-reporting by each responding department director.

We recognize social desirability as a concern for survey responses. The survey specially asks for change in use rather than rate of use for the two years to avoid anchoring bias or halo effect between years. This perception of use by managers was important to consider given the interest in performance management beyond performance measurement. While we recognize the concern of bias, variation in results including those reporting limited change lessen that this effect is prominent in the data.

### Sample

We collected survey data from six municipal departments (budgeting, human resources, inspections, parks, planning, and public works) in North Carolina cities and towns with populations greater than 10,000 (90 municipalities qualified for inclusion based on size). The specific departments represent fundamental core services across surveyed municipalities. They also offer a mix of internal and external-facing functions. The population size of the municipalities increased the probability that their workforces would be large enough to have dedicated staff within these departments.

Email addresses for department heads were assembled from municipal websites. The survey was administered to 472 department directors. Some municipalities lacked the departments within their organizations completely, especially for the smaller jurisdictions. Municipalities that contracted services to the county or had consolidated departments were excluded. Some websites did not provide contact information for departmental director resulting in them being excluded from the final sample. We received 103 responses (21.82% response rate). Table 1 shows the distributions of respondents by department.

**Table 1.** Survey respondents.

Department	Number
Budgeting	21
Human Resources	19
Inspections	13
Parks	14
Planning	21
Public Works	15
Total	103

### Analysis

To understand what changes municipalities made to the level of performance management use during the COVID-19 pandemic, we used descriptive statistics to compare performance management analysis and use in 2019 (pre-pandemic) and 2020 (pandemic) across the full sample. Next, we examined the changes by department type to determine whether there was a variation in how different local government departments changed their performance management practices during the crisis.

An Ordinary Least Squares (OLS) regression analysis was used to determine the changes in performance management practices during the pandemic. This helped address the second research question of this study, which examines whether pre-pandemic performance management use affected use levels during the pandemic. Equation (1) represents the estimation model.

(1) $$\Upsilon_{i}=\beta_{0}+\beta_{1}\Psi_{i}+\beta_{2}\nabla_{i}+X_{i}\Gamma +\varepsilon_{i}.$$

$\Upsilon_{i}$ represents the change in performance management during the pandemic for department i. The dependent variable is thus the response to the survey that asked participants to indicate on a sliding scale of 1 to 7 the extent to which performance management use had changed between 2020 and 2019 (See Appendix A). $\beta_{0}$ is the constant and $\beta_{1}$ is the coefficient representing the relationship between the pre-pandemic status of performance management ($\Psi_{i}$) and $\Upsilon_{i}$. $\nabla_{i}$ is the dichotomous variable for whether a department is budgeting and finance or not, and $\beta_{2}$ represents the coefficient for the difference in changes made by budgeting and finance departments compared to all other departments.

$X_{i}$ represents the matrix of two control variables: the extent to which the department adheres to formal decision-making and changes in service demand during the pandemic. Given the small size of our sample we were limited on the number of control variables. We ran models that included organizational size and tenure of respondents, but they were not statistically significant. We were unable to determine political stability. $\varepsilon_{i}$ represents the unobserved random error, clustered by city.

## Results

### Performance information analysis

**Figure 1.**
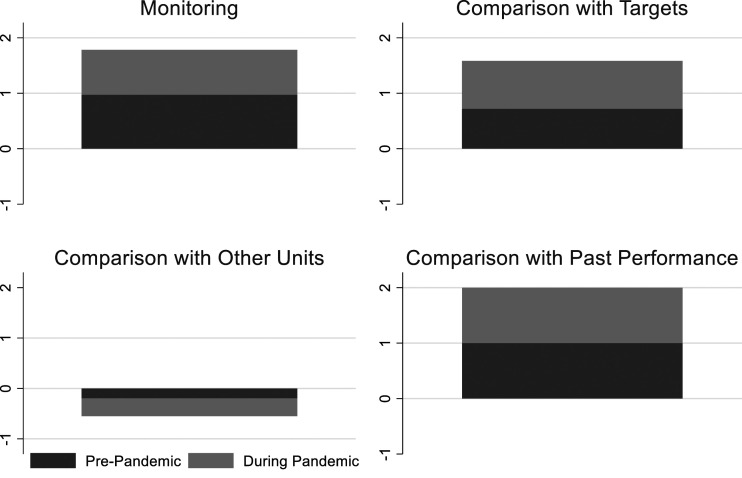
Change in Performance Information Analysis During the Pandemic.

Respondents indicated an increase in analysis of performance information during the pandemic (2020) compared to 2019 for all measured areas except as it relates to conducting comparisons with other units (benchmarking), which showed a decrease (see Figure 1). Since the 2020 response variables denote a measure of ‘change’ from the base year, a negative value for the dependent variable means a decrease in relation to the base year (2019), whereas a positive value means an increase.

Directors reported that even before the pandemic (2019), their departments engaged in benchmarking less than the other (internally focused) forms of performance information analysis—including monitoring, comparison with targets, and comparison with past performance—and the use rate declined further in 2020. It may be that if the practice and culture of using data drawn from comparing to external peers is not established before a crisis, the time-consuming process of developing benchmarks, collecting data, and properly analyzing the results are less likely to be initiated during a crisis. It’s also likely that individual department needs and expectations were changing so rapidly, especially in the early stages of the pandemic, that determining and stabilizing internal practices was a challenge let alone establishing comparison with external jurisdiction who were also in flux.

Municipal departments reported an increased use of performance information during the pandemic (2020) to monitor progress and compare it with internal targets and past performance. This increase is understandable since performance information gives public managers a better idea about the current situation, how far they are from achieving their goals, and how much they have deviated from the previous performance (Bidya 2009; Behn 2003; Grant 2003) — performance information that likely became even more important during the pandemic. As jurisdictions struggled to determine how to respond in uncertain times internal analysis could help to inform choices and support hard decisions.

**Figure 2.**
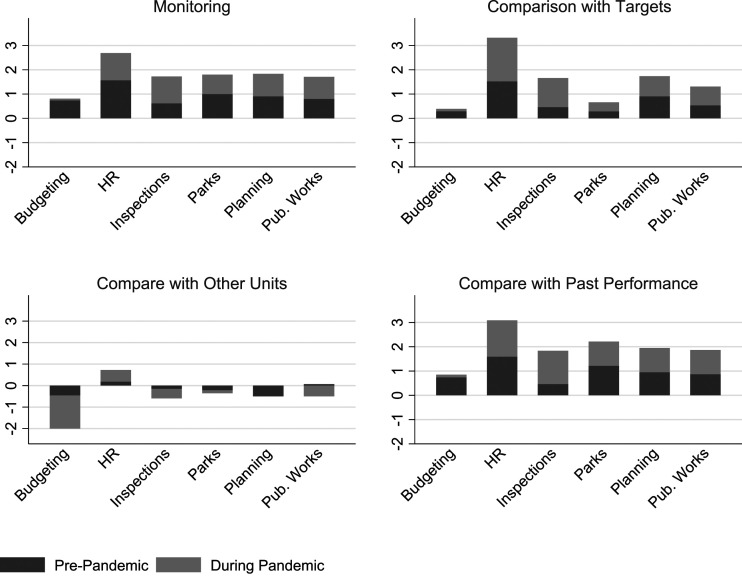
Change in Performance Information Analysis During the Pandemic by Department.

Though the usage rates vary substantially, the trend is consistent across department types, except for budgeting departments (see Figure 2). In general, the various departments engaged in increased levels of performance information analysis as it related to monitoring performance information and comparing it with targets and past performance. The budgeting and finance departments, however, showed the smallest increase in performance information analysis and the biggest decrease in comparison with other measures during the pandemic. One possible reason for this is that, despite many budget departments facing substantially decreased budgets and enhanced workload to have to revise budget projections and associated communications, the overall budgeting process remained stable.

Human resources was the only department that increased the use of benchmarking. Given that local governments were under tremendous pressure to figure out how to manage a workforce in a crisis while moving large numbers of employees to remote work (Delfino and Van der Kolk 2021), it makes sense that human resources departments wanted to know how their colleagues in other municipalities were responding.

### Performance information use

**Figure 3.**
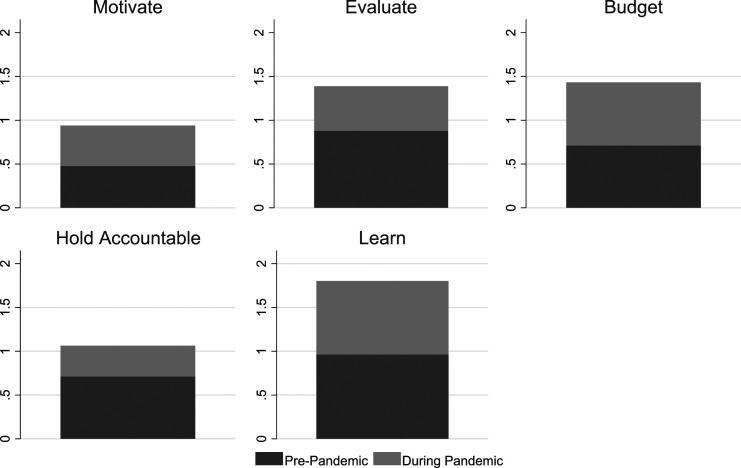
Change in Performance Information Use During the Pandemic.

Performance information use refers to the practice of making decisions and taking action based on the information generated by a performance measurement system (Moynihan 2008; Poister, Aristigueta, and Hall 2014). Overall, the surveyed municipalities reported an increased use of performance information focused on motivation, evaluation, budgeting, accountability, and learning during the pandemic (2020) (see Figure 3). Not surprisingly, the use of performance information for the purposes of motivation, evaluation, and accountability increased less than for budget and learning. With economic challenges hitting municipalities hard, budgets had to be substantially revised. The biggest increase was seen in the use of performance information for learning. The immense changes caused by COVID-19 required constant learning—perhaps one of the more encouraging consequences of the pandemic.

**Figure 4.**
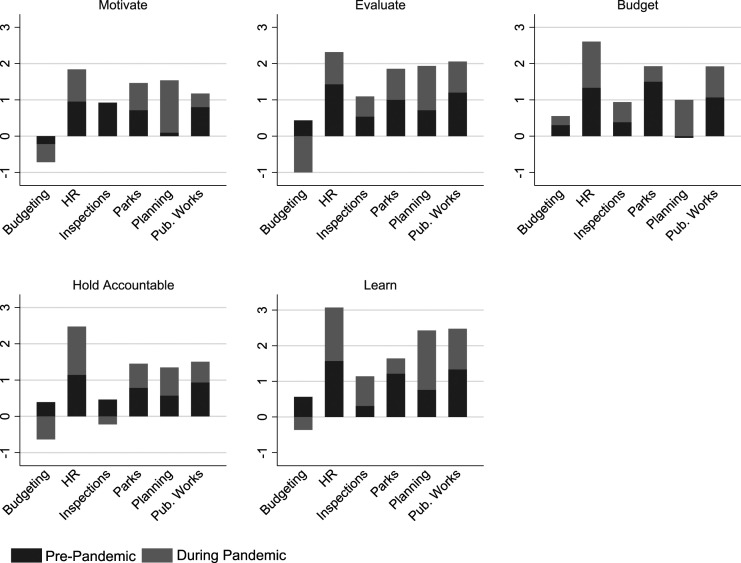
Change in Performance Information Use During the Pandemic by Department.

Across departments, the pattern of enhanced use remained consistent except for budgeting departments (see Figure 4), which reported a lower-than-average use of performance information for motivation both before and during the pandemic. Given the economic downswing that accompanied the pandemic, many budget and finance offices were scrambling to redo their budgets and make massive, unplanned cuts. Their priority was financial data—not data for managerially efforts. Though this lack performance information designed and collected to inform and improve managerial efforts on motivation is an area for improvement for these offices that will need to not only motivate but worry about retention of talented public servants.

The departments of inspections and permits used performance information for employee motivation with the same intensity as the prior year but decreased their use of performance information focused on holding employees accountable. Given the changing nature of work during COVID-19 (especially early in 2020), accountability measures may not have been as viable as in previous years—with industries putting projects on hold plus the problem of safely conducting in-person inspections during a pandemic, many inspectors simply couldn’t complete their assignments. In the pre-pandemic period, the departments of planning and economic development made limited use of performance information for employee motivation or budgeting decisions, but they dramatically increased their use of it during the pandemic (2020).

### Stakeholder interest in performance information

**Figure 5.**
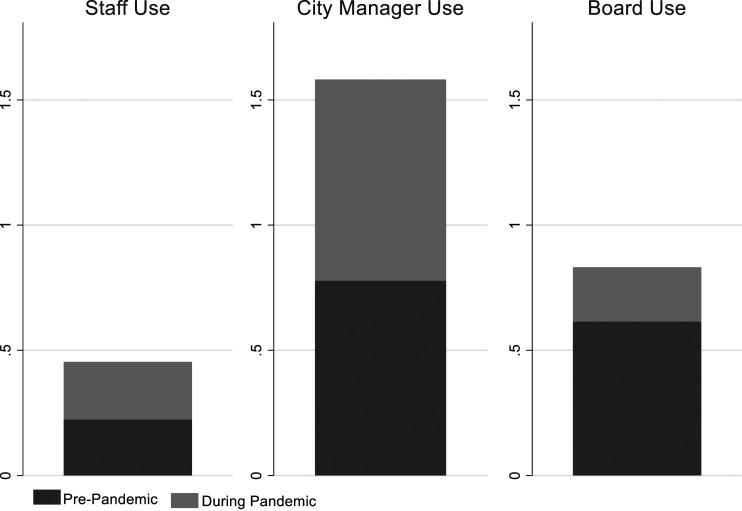
Change in Performance Information Use by Stakeholders During the Pandemic.

Performance information usage rates are an important part of the picture of performance management within local governments, and it’s also important to better understand who is tracking, using, and interested in the data. Department heads reported an increased use of performance information by line department staff, city managers or management teams, and elected boards in 2020 compared to the previous year (see Figure 5). While all groups saw an increase, the levels before and during the pandemic varied. Respondents reported the greatest increase for this survey item: “Our city manager/management team regularly monitors our performance measures.”

Performance information use at the staff level (anyone below the director) allows employees to better understand where the organization is headed and whether their strategies are working. For this analysis, we looked at the question of staff use by asking directors the question: “My staff frequently uses performance measures in proposals and requests they make to me.” Directors reported little staff use of performance information for this purpose in 2019 and only a small increase in 2020.

As noted above, the department heads indicated that their city managers monitored performance information more frequently during the crisis (2020). The increase could have been driven by their need to understand where the department was headed and what decisions had to be made as well as to respond to board and citizen requests for information. It also could have been a result of city managers’ need to make complex decisions across departments that had competing service needs.

The directors indicated that board interest in performance information during the pandemic increased, likely a consequence of board members’ need to respond to an array of concerns expressed by citizens and a desire to feel an increased level of understanding during very uncertain times.

The directors of all departments except budget departments indicated that staff use of performance measures in proposals and requests increased at varying levels during the pandemic. In 2019, budget offices were already infrequent users of performance information for this purpose, and they used it even less in 2020, likely because of their intense focus on financial matters and standardized budget processes. The budget directors in the survey reported that board interest in their performance information also declined, though the monitoring of their performance information by city managers increased in 2020.

The political pressure on elected officials during the pandemic was high. As thorny issues of mask mandates and office, business, and school closings took center stage, some departments invariably shifted away from internal data. Directors of human resources, inspections, and, to a lesser degree, parks departments did acknowledge increased interest from board members in their performance information. All departments with the exception of parks (most parks were closed during this time) saw their city managers increasing the extent to which they monitored performance data.

Overall, with few exceptions, the directors indicated that other stakeholders showed an increased interest in and use of performance information during the pandemic (2020) compared to the previous year. As hard decisions were being made in all departments, directors of human resources consistently indicated the greatest increase, which was not unexpected given the workforce changes in 2020 and the need to design and monitor new approaches to work.

### Regression analysis

**Figure 6.**
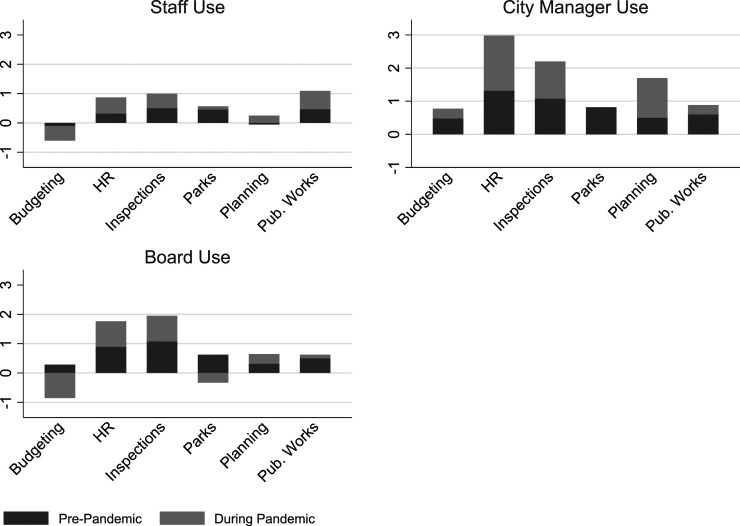
Change in Performance Information Use by Stakeholders During the Pandemic by Department.

Consistently, municipal departments reported increased levels of performance information analysis and use during the pandemic (2020) compared to the previous year (see Figures 1, 2, 3, 4, 5 and 6). What factors could explain these changes? To address this question, we examined OLS regressions estimates with a focus on the extent to which prior performance management practices may help to explain pandemic practices.

Table 2 shows the relationship between reliance on performance management practices before and during the pandemic. This analysis was done for both use and analysis measures to provide a more complete view of changes to performance management practices. Results indicate that one additional unit of pre-pandemic performance monitoring is associated with a 0.320 unit increase in the activity during the pandemic. Similarly, one additional unit of comparison with targets and other agencies before the pandemic is associated with 0.361 and 0.753 unit increases in these practices during the pandemic, respectively. Goals setting, identification of appropriate comparison groups, and collection all require careful analysis and planning. When such systems are in place it appears that they are being leveraged by the departments that have invested in them while those that have not are less likely to divert limited resources to developing these. While the trend for a number of years has been increased adoption and use of performance management systems it appears that the pandemic served to limit this trend as it did with many other initiatives and municipal strategic priorities.

**Table 2.** OLS Regressions Estimating the Relationship between Performance Management Practices prior to and during the COVID-19 Pandemic.

	Monitor	Compare w/Target	Compare w/Others	Compare w/Past	Motivate	Evaluate	Budget	Hold accountable	Learn	Staff use	City manager use	Board use
	(1)	(2)	(3)	(4)	(5)	(6)	(7)	(8)	(9)	(10)	(11)	(12)
Pre-Pandemic Status	0.320*	0.361**	0.753***	0.181	0.647**	0.624**	0.462**	0.548**	0.524**	0.716***	0.789***	0.922***
	(0.168)	(0.142)	(0.170)	(0.196)	(0.248)	(0.247)	(0.214)	(0.239)	(0.210)	(0.177)	(0.191)	(0.189)
Formal Strategy	−0.174	−0.061	−0.484	−0.169	0.073	0.195	0.313	0.254	0.361	0.408	0.284	0.323
	(0.206)	(0.207)	(0.291)	(0.266)	(0.236)	(0.237)	(0.286)	(0.261)	(0.262)	(0.295)	(0.217)	(0.303)
Service Demand	−0.187	−0.089	−0.081	−0.121	0.156	0.166	0.471*	0.221	0.309	−0.073	0.130	−0.139
	(0.190)	(0.173)	(0.202)	(0.233)	(0.225)	(0.211)	(0.255)	(0.216)	(0.276)	(0.230)	(0.231)	(0.254)
Budget Dummy	−1.130***	−0.879**	−0.849	−0.968	−0.321	−1.181**	0.488	−0.505	−0.409	0.216	−0.066	−0.018
	(0.328)	(0.376)	(0.714)	(0.577)	(0.585)	(0.545)	(0.639)	(0.538)	(0.623)	(0.482)	(0.517)	(0.702)
Constant	1.021***	0.865**	0.049	1.227**	−0.182	−0.160	−0.464	−0.236	−0.053	0.094	−0.216	−0.072
	(0.358)	(0.375)	(0.415)	(0.497)	(0.544)	(0.528)	(0.579)	(0.511)	(0.605)	(0.529)	(0.544)	(0.599)
Obs.	69	58	56	53	53	50	52	49	48	49	50	37
R-squared	0.147	0.185	0.256	0.072	0.282	0.385	0.237	0.302	0.300	0.373	0.411	0.529

Note: Unstandardized coefficients; Robust standard errors in parentheses; ***p < 0.01, **p < 0.05, *p < 0.1.

Municipal departments that used performance information to motivate employees before the pandemic were likely to increase this practice by 0.647 units during the pandemic. One higher unit of pre-pandemic use of performance information to evaluate, budget, hold accountable, and learn is associated with a 0.624, 0.462, 0.548, and 0.524 unit increase during the pandemic, respectively. One additional unit of pre-pandemic use of performance information by staff is associated with a 0.716-unit increase, whereas one additional unit of the perceived city manager and board use is associated with a 0.789 and 0.922 unit increase each during the pandemic. Organizations that have done the work to consider the managerial purposes, associated performance measures, collection and use are more likely to continue to deploy these measures and use them in times of intensified decision making as was marked by managing in the midst of the pandemic.

Additionally, these findings support scholarship (Ammons 2020; Kroll and Vogel 2021) that highlight the importance of increased interest by stakeholders of performance management systems. If stakeholders are practiced in using and relying on performance information the probability of use of such information in times of crisis or confusion will increase. A crisis is not a time to learn how to request, apply, and interpret information that is not part of the established decision-making models.

## Discussion

Municipal departments reported an increased use of internally focused performance information during the pandemic (2020). While this exploratory study does not evaluate the effectiveness of using increased information previous scholarship would indicate that such practices could prove to enhance the decisions being made. Performance management systems have been argued to enable managers to use performance information to improve results, including service quality and efficiency (Ammons and Roenigk, 2015). As such the hope is that the increased use of performance information during the pandemic could service to assist in critical decision making.

We find that organizations tend to fall back on their existing capacity and work with systems they are most comfortable, hence municipal department that had developed previous practices could leverage them (Kim and Chen 2020). The influence of pre-pandemic stakeholder use was strong on post-pandemic performance management, which likely speaks to the notion that having an established system of performance management in place—one in which employees and board members use data to make decisions—allows those tools to be leveraged during a crisis. These findings are in line with the expectations of institutional and path dependent theories that depict a more conservative view of organizations reliant on an interaction of nested rules, exclusive membership, and power arrangements (Hodgson 2006; North 1990).

Indeed, fundamental shift in decision-making practices that are rooted in organizational culture can be exceptionally challenging during the best of times and even more daunting in a time of crisis that often involves increased pressure on resources and enhances employee resistance (Bovey and Hede 2001; Bordia et al., 2006; Peters, Pierre, and Randma-Liiv 2011; Sargiacomo et al., 2021; Thelen 2000). Hence, our findings do not find significant change in decision-making methods and the associated data use during the COVID-19 pandemic (also see Thelen 2000). As scholarship continues to recognize, performance management must become part of the cultural fabric of an organization for it to truly move from measurement to management system. Moreso, the existence of a performance management culture appears to be critical for continued or increased performance management use during crisis or stressful organizational times.

While it could be expected that with increased or altered service demand, departments might rely more on budget performance information for resource based decision making, we did not observe this behavior within our sample. With so many changes made to how services were delivered, drawing on measures designed for comparison with the past or others would likely not serve to inform decision making against others (a practice that saw a limited increase in the pandemic) was largely influenced by past behavior.

The results found that the extent that a municipal department has strategy development that is more formalized had a negative (though not significant) relationship for analysis measures but positive for both use and stakeholder use measures. These trends, though not significant, warrant future attention. The notion that the development of strategic plans and formalized strategy are fundamental to the operating of a performance management system appears to be one that continues to come into question leaving critical questions in need of more empirical scholarship (Ammons and Roenigk, 2015).

Across the analysis it was found that departments act differently in their use of performance information with the Budget Department being an interesting outlier as seen in both the descriptive and regression results. Returning to Behn’s premise that the purpose for the design, collection, and use of measures needs to be forefront it raises questions as to how different departments consider the value and use of performance information, how it becomes part of their managerial decision-making, and why budget departments do not indicate the use nor interest in engaging in common sources of performance information (2003).

These findings also raise questions as to if departments may be reverting to less desirable performance management practices such as we would expect from the principal-agent theory in which data may be used to for accountability purposes. Though we have no empirical reason to believe this is happening within the sample, it is important to recognize that while contemporary scholarship has moved away from these accountability-oriented approaches, that less desirable approach to data use and presentation is still a real concern as indicated in principal-agent theory research. Research and practice needs to remain diligent in ensuring that performance data is used appropriately and not in ways that would provide false or inaccurate information and harmful management responses.

We acknowledge that these findings could reflect response bias, with organizations making more use of performance management being more likely to respond to our survey. These findings may also demonstrate Matthew Effects (see Merton 1968), in the sense that those who already used performance management are placed in situations where they make more use of these systems, whereas others struggle to implement them.

## Conclusion

In this study, we set out to explore two pressing questions regarding changes in public management during a crisis. First, what changes did municipalities make to the level of use performance management during the COVID-19 pandemic? Second, what factors impacted performance management changes during the pandemic? In general, we saw that public managers reported a higher analysis and use of performance information in 2020 compared to 2019. Our findings support previous work showing that crises like COVID-19 and the Great Recession force organizations to change the way they use performance management systems.

Municipal departments that reported a higher pre-pandemic analysis and use of performance information were more likely than others to intensify their reliance on these practices during the pandemic. This finding supports the assertion that changes, however transactional or transformational they might seem, emerge only out of the limited options that an organization’s past decisions and existing structure allow (Hodgson 2006; North 1990; Pierson 2000). A crisis may prevent truly large-scale changes to the internal structure and operations of organizations, but it can encourage them to build upon and leverage their existing capacity (Peters, Pierre, and Randma-Liiv 2011; Thelen 2000). Regardless of the effectiveness of performance management systems in mitigating the crisis, municipal departments with existing performance management capacity were more likely to rely on this practice during the pandemic.

The ongoing pandemic provides an opportunity for public management scholarship to revisit its theoretical and empirical foundations to enhance our understanding not just of this pandemic but of other deep-seated and insidious problems in our workplaces (Dunlop, Ongaro, and Baker 2020; Pollitt 2016). This study is one step toward making such progress. Our findings suggest that public managers do change their behaviors and practices to adjust and respond to crises, even if those changes are rooted in existing and prevalent norms and systems. And when managers develop and sustain critical systems during times of stability, they are able to leverage those systems during a crisis to deal with extreme uncertainty.

This exploratory research aims for analytical generalizability, not statistical generalizability, and has a limited sample. It highlights the need for further research and sets up potential questions for exploring how data is used in a crisis, how effective the decisions are that rely on it, and how its use influences the behavior of our public servants. We invite future research to include larger sample size and more robust empirical methodology to inform and expand on our collective knowledge of these and related questions.

## Note

1. *See* “A Timeline of Covid-19 Developments in 2020,” *The American Journal of Managed Care* (AJMC), https://www.ajmc.com/view/a-timeline-of-covid19-developments-in-2020.

## Appendix A.

**Appendix A.** Survey items.

Focus area	Item
Analysis	Please indicate your level of agreement on a scale of 1–7 with the following statements as they apply to 2019 (pre-COVID).
	• I regularly monitor our performance data.
	• I regularly compare our performance measures against our department targets.
	• I regularly compare our performance measures against other similar departments in other jurisdictions.
	• I regularly compare our performance measures against our own past performance.
	• Please indicate how, if at all, engagement in these practices changed during COVID (2020) by moving the slider scales (1-10) below.
	• I regularly monitor our performance data.
	• I regularly compare our performance measures against our department targets.
	• I regularly compare our performance measures against other similar departments in other jurisdictions.
	• I regularly compare our performance measures against our own past performance.
Use	Please indicate your level of agreement with the following statements as they apply to 2019 (pre-COVID).
	• I regularly used performance data to motivate my subordinates.
	• I regularly used performance data to evaluate my department’s performance.
	• I regularly used performance data to budget programs and projects.
	• I regularly used performance data to hold my subordinates accountable.
	• l regularly used performance data to learn what is working and what we need to do differently to improve.
	Please indicate how, if at all, engagement in these practices changed during COVID (2020) by moving the slider scales below.
	• I regularly used performance data to motivate my subordinates.
	• I regularly used performance data to evaluate my department’s performance.
	• I regularly used performance data to budget programs and projects.
	• I regularly used performance data to hold my subordinates accountable.
	• I regularly used performance data to learn what is working and what we need to do differently to improve.
Stakeholder Interest	Please indicate your level of agreement with the following statements as they apply to 2019 (pre-COVID).
	• My staff frequently uses performance measures in proposals and requests they make to me.
	• Our city manager/management team regularly monitors our performance measures.
	• Our board shows great interest in our performance information.
	Please indicate how, if at all, engagement in these practices changed during COVID (2020) by moving the slider scales below.
	• My staff frequently uses performance measures in proposals and requests they make to me.
	• Our city manager/management team regularly monitors our performance measures.
	• Our board shows great interest in our performance information.
Demand	Compared to 2019 (pre-COVID), the demand for our department’s services in 2020 (during COVID) was:
	• Much Lower to Much Higher (7-point scale)
Formal Strategy	Please indicate where you would place your department in terms of strategy development on the continuum below (by moving the slider scale with 0 representing informal and 10 representing formal strategy development):
	• 2019
	• 2020

**Appendix B.** T-test of responder versus non-responder.

Responder status	Obs.	Mean of population	
No	271	58,982.37	t = −0.5623
Yes	207	65,294.31

**Appendix C.** Responder versus non-responder by departments.

	Budgeting	Human resources	Inspections	Parks	Planning	Public works	Total
Responder status							
No	42	36	49	47	50	47	271
Yes	39	39	30	35	33	31	207
Total	81	75	79	82	83	78	478
